# Azide click chemistry on magnetotactic bacteria: A versatile technique to attach a cargo

**DOI:** 10.1016/j.mtbio.2023.100587

**Published:** 2023-02-23

**Authors:** Paul Eduardo David Soto Rodriguez, Mila Sirinelli-Kojadinovic, Maximilien Rouzaud, Damien Faivre

**Affiliations:** Aix Marseille University, CEA, CNRS, BIAM, 13108 Saint Paul-Lez-Durance, France

**Keywords:** Click chemistry, Magnetotactic bacteria, Single-cell tagging

## Abstract

Adding biomolecules to living organisms and cells is the basis for creating living materials or biohybrids for robotic systems. Bioorthogonal chemistry allows covalently modifying biomolecules with functional groups not natively present under biological conditions and is therefore applicable to microorganisms and cells. Click chemistry is a biorthogonal chemistry approach that allows the study and manipulation of living entities. Incorporating the bioorthogonal click-chemistry handle, azide groups, into living microorganisms has been achieved by metabolic labeling, i.e., by culturing cells or organisms in a modified culture media having a specific natural molecular building block (e.g., amino acid, nucleotide, carbohydrate) modified with a tagged chemical analog. Here we explore the effect of the azide group incorporation into the magnetotactic bacteria *Magnetospirillum gryphiswaldense* (MSR-1) by adding a modified amino acid, 3-Azido-d-Alanine, during their cultivation. We show the existence of a concentration limit to effectively incorporate the azide group while maintaining the magnetic properties of the cells. We explore the use of this modification to explore the combination with versatile single-cell tagging methods.

## Highlights

• A high concentration of modified amino acid inhibits magnetosome production by magnetotactic bacteria.
• An optimal concentration range of modified amino acid maintains bacterial magnetic properties and allows the incorporation of azide groups efficiently.
• Copper-free azide click chemistry is feasible for functionalizing magnetotactic bacteria.
• The process is compatible with other standard bacterial staining procedures.

## Graphical abstract

**Figure:**
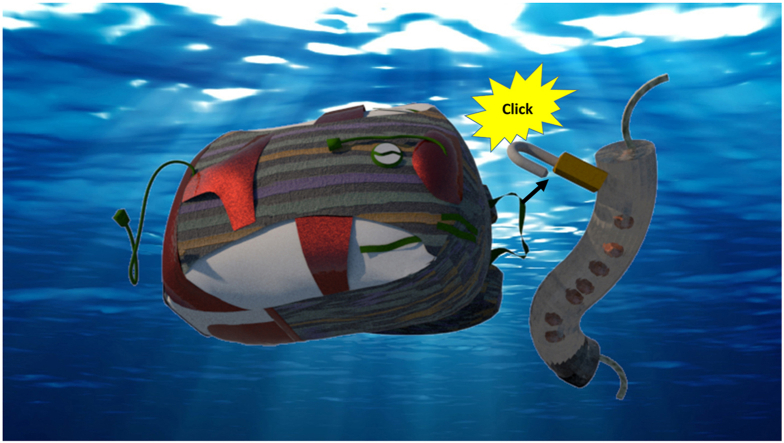

## 1 Introduction

Theranostics is one of the main application field of nano and micro-robotics [1]. Many designs developed for theranostics are based on mimicking microorganisms, particularly introducing motility and sensing functionalities [2]. Magnetic properties often provide or at least improve theranostic applications. Therefore, the attention on magnetic nanoparticles is rising in this area [3]. Another rising research field is the so-called bacteria bots, which studies the direct modification of bacterial surfaces to attach a cargo that can be used for detection and treatment of a given illness [4]. Different bacterial strains and cargo designs have been proposed [[5], [6], [7], [8]]. The primary desired characteristics are that bacteria should allow easy attachment of biomolecules, be able to control and provide easy characterization remotely. Many bacteria have been genetically engineered or chemically modified to fulfill these properties, including nanoliposomes attachment through amine coupling by activation of carboxylic acids with EDC/NHS chemistry [9], by NHS-ester amine coupling [10] or biotin-streptavidin reactions [11].

Magnetotactic bacteria (MTB) mineralize and organize intracellularly magnetic particles called magnetosomes. The latter allow them to swim along the geomagnetic field lines [12] and therefore enable magnetic control of MTB motility by external magnetic fields. In addition, MTB use a magnetically-biased aerotaxis that allows them to find their preferred living conditions [13]. This particular behavior has been used to develop active systems envisioned to deliver drugs to cancer cells [9,14]. Moreover, the magnetic properties of magnetosomes are used as a contrast agent in biomedical imaging applications. For example, for magnetic resonance imaging (MRI) when isolated [15], or as tracer particles in magnetic particle imaging (MPI) isolated [16] or even within the cell [17].

**Scheme 1:**
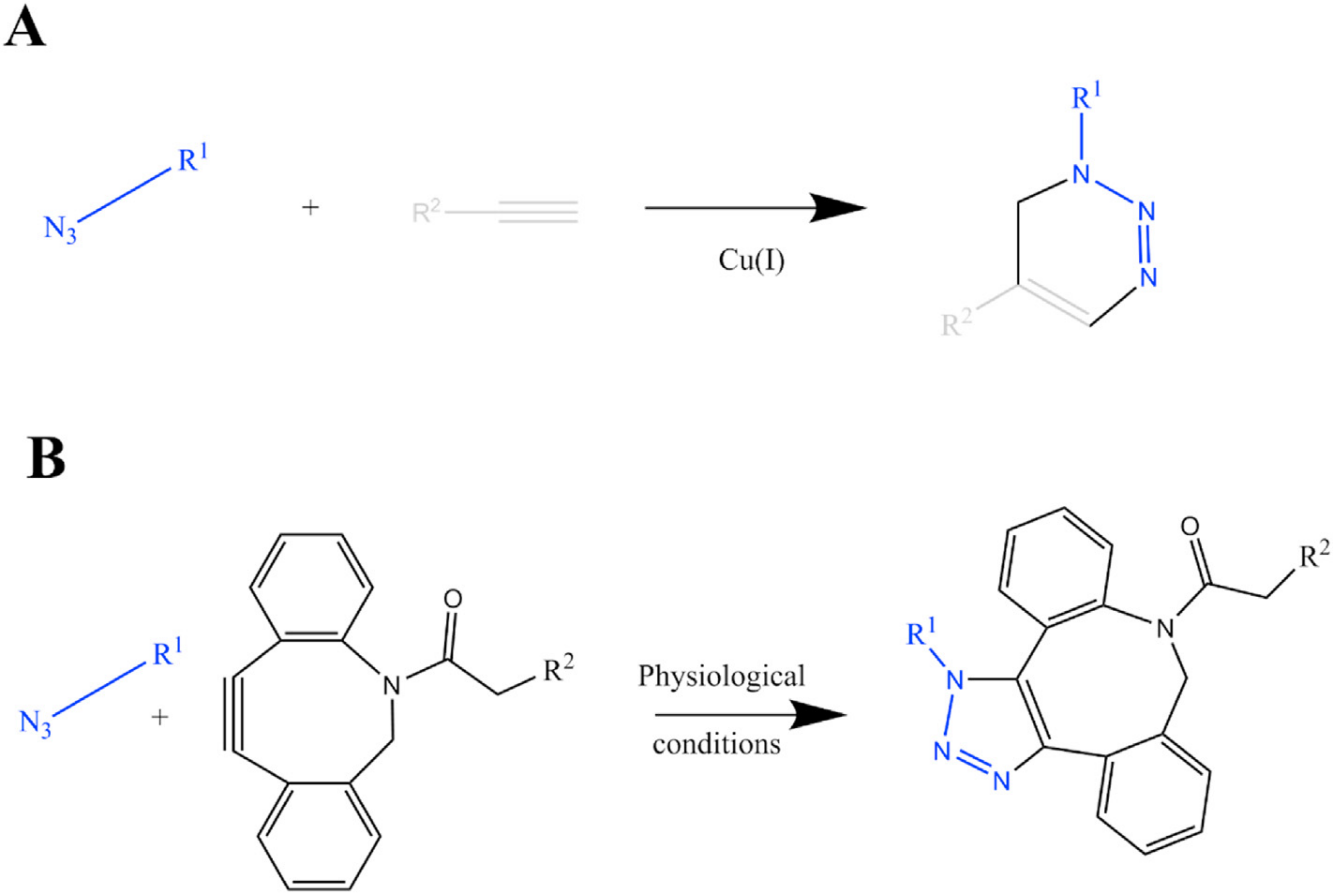
Click chemistry. A) Copper(I)-catalyzed azide alkyne cycloaddition (CuAAC) mechanism. B) Copper free click chemistry with the strained Dibenzo cyclooctene (DBCO) molecule.

Click chemistry, topic for which the 2022 Nobel prize awards were given [18], refers to a set of fast reactions, easy to use, simple to purify, versatile, regiospecific, and with high product yields [18]. Click chemistry permits different biomolecules’ easy and fast attachment to particles. The group of reactions mainly used is the Huisgen 1,3-dipolar cycloaddition of azides and terminal alkynes. To date, it has not been hardly exploited for its use in magnetotactic bacteria. Indeed, the application of alkyne-azide click chemistry is not straightforward as the needed functional group azide is not naturally present in bacteria. Moreover, as shown in Scheme 1 A, the conventional azide-alkyne binding is done with the help of copper(I) as a catalyst [19], which, in general, is harmful to bacteria at the concentration used [20]. However, there are methods to overcome these drawback. For example, azide groups can be incorporated into living animals by genetic engineering [21,22] or metabolic labeling and can therefore enable copper-free click chemistry to label biomolecules in mice [23]. In addition, copper-free strain-promoted azide-alkyne cycloaddition has already been developed and used as an alternative in living cells and was recently awarded with a Nobel prize 2022. For this method, a strained molecule (Dibenzocyclooctyne, DBCO) is used that can react with the azide without the need of the copper catalyst as show in Scheme 1B.

In the present work, we describe azide group incorporation by bacteria. This will pave the way for future click chemistry on bacterial surface, providing the necessary engineering tools to modify bacteria as needed with the reported advantage of MTB in terms of control and theranostics.

More precisely, we propose to couple MTB and copper-free strain-promoted azide-alkyne cycloaddition to join the advantages of these two separate entities. We thus feed MTB with a modified amino acid, 3-Azido-d-alanine, to enable click chemistry on their surface. The unnatural d-amino acids are incorporated into cell walls; the bioorthogonal (azide) group is cut and integrated into the surface [24]. We investigate bacterial physiological properties to determine how the incorporation of the modified amino acid impacts (cell growth, biomineralization capabilities and hence magnetic properties). We show that amino acid concentration must be controlled to avoid stressing bacteria. Finally, we show successful click chemistry by adding different fluorescent chemical compounds but also 10 ​μm-beads as cargos. These results will enable the facile chemical engineering of MTB for a given application.

## 2 Materials and methods

### 2.1 Materials

DBCO-PEG4-FLUOR 545; DBCO-Cy5; DBCO-dPEG ®12-carboxyfluorescein were purchased from ***Sigma Aldrich***, while the 3-Azido-d-Alanine Hydrochloride was purchased from ***BASECLICK***. Micromer®-DBCO 10 ​μm was purchased from ***micromod Partikeltechnologie GmbH***.

### 2.2 Methods

#### 2.2.1 Bacterial cultivation and incorporation of the modified amino acid

*Magnetospirillum gryphiswaldense* (MSR-1) cells were grown at 28 ​°C under microoxic conditions) in Hungate tubes containing 12 ​mL of modified Flask Standard Medium (FSM) containing 50 ​μM ferric iron citrate [25]. Bacterial cultures were started at low cell density, i.e., at OD (600 ​nm) ​≅ ​0.009 to 0.02, from pre-cultures in mid-stationary phase and were grown for three days. To effectively incorporate the amino acid and use the cells for further modification, the modified amino acid was added directly after bacterial inoculation. Three concentrations of 3-Azido-d-alanine were tested: 0.009 ​mM, 1.080 ​mM, and 1.800 ​mM.

#### 2.2.2 Methods used to assert the effect of azide incorporation on the MTB magnetic properties

A customized magnetic microscope was used [26]. Briefly, the microscope has a triaxial Helmholtz coil set, a controller (C-SpinCoil-XYZ, Micro Magnetics Inc.) and an Andor Zyla 5.5 high-speed camera. The 3D-axis Helmholtz coils generate D.C. magnetic fields with a precision of 5% of the Earth's magnetic field (2.5 ​μT, manufacturer's specifications). The magnetic field was switched by applying −3.5 and ​+ ​3.5 ​mT for the so-called U-turns, programmed at different time steps. The magnetic field values were fixed for 2 ​s before the switching value, and the switching was repeated over a fixed time-lapse, ensuring the observation of at least three U-turns in the field of view. The trajectories and U-turns were extracted and smoothed by a tracking script written in python and based on the OpenCV Object Tracking Algorithms with the CSRT tracker [27]. A convolution-based smoothing approach smooth the data. For the U-turn data, 12 ​cells from each condition were analyzed. A 40× objective (Nikon Apochromatic Lambda S 40× WI, N.A. 1.15, water immersion) was used for both trajectory and U-turn measurements. The mathematical relation to calculate the magnetic moment from the U-turn is the one used by C.J. Pierce et al. [28] valid for low field as those used in this study with the drag coefficient modification using the prolate ellipsoid approximation as done by P. Leao et al. considering length and width of the cels [29]:

$$\tau_{Uturn}=\frac{f_{r}}{M_{cell}∙H}=\frac{16∙\pi ∙ɳ∙A^{3}}{3∙M_{cell}∙H}$$

Here, ***τ***_***Uturn***_ corresponds to the time the MTB takes to make a U-turn upon switching the magnetic field (***H***); ***ɳ*** corresponds to the media viscosity; $M_{cell}$ to the magnetic moment of MSR-1 ​cell; ***A*** is defined as:

$$A=\frac{c^{3}}{\frac{1}{2}\left[\left(\frac{a+c}{a-c}\right)-\left(\frac{a∙c}{b^{2}}\right)\right]}$$

With a and b being the major and minor semi-axis respectively of the prolate ellipsoid and $c=\sqrt{\left(a^{2}-b^{2}\right)}$;.The MSR-1 U-turn time (***τ***_***Utur***n_) was determined by tracking the U-turn trajectory and then plotting its first derivate, corresponding to the instantaneous velocity. Note that the change in sign corresponds to the change in direction. The value of ***τ***_***Uturn***_ is obtained by taking the time difference between the two steps as described in the section results and discussion, such that $M_{cell}$ can then be calculated from equation (1).

$M_{cell}$. All data fitting was done by the intrinsic fitting functions of OriginPro [30].

#### 2.2.3 Fluorescent tagging efficiency

A semi-quantification analysis was done with a custom python script. The script extracts the fluorescence labeled MTB by converting the images to HSV and extracting the colored (fluorescent) layer to a new image and counting the amount of labeled MTB. The total amount (tagged and non-tagged) of MTB in the images are obtained by converting the image to gray scale and improving the image contrast with an adaptive histogram approach it then calculates the outline with a canny edge detector algorithm and finally the total outlines obtained were then counted. A relative error was then defined as total amount of MTB minus number of tagged MTB divided by the total amount of MTB and converted to percentage by multiplying by 100. This relative error was then considered as a parameter to compare the tagging efficiency.

#### 2.2.4 Click chemistry

DBCO-PEG4-FLUOR 545 a.k.a. DBCO-PEG4-TAMRA (DBCO-TAMRA), was used to show the presence of the azide group after incorporating the modified amino acid. MSR-1 ​cells grown with the amino acid during 3 days (O.D (600 ​nm) ​= ​0.2) were re-suspended in PBS (0.1 ​mM; pH 7.4) and mixed with 100 ​μM of DBCO-TAMRA for 1 ​h while shaking at 700 ​rpm under dark conditions and at room temperature. The resulting solution was washed 3 times with PBS and centrifuged at 3500 ​g. For imaging, MSR-1 was left in PBS. For motility measurements, bacteria were re-suspended in FSM.

The attachment of the 10 ​μm, Micromer®-DBCO beads was done by adding 2 ​mg/mL of beads to a 2 ​mL solution of MTB (MTB-GFP) in PBS with an OD around 5.0 and left for 1 ​h while shaking at 700 ​rpm under dark conditions and at room temperature. The resulting solution was washed 3 times with PBS and centrifuged at 3500 ​g. For imaging, MSR-1 was left in PBS. For motility measurements, they were re-suspended in FSM.

## 3 Results and discussion

**Fig. 1:**
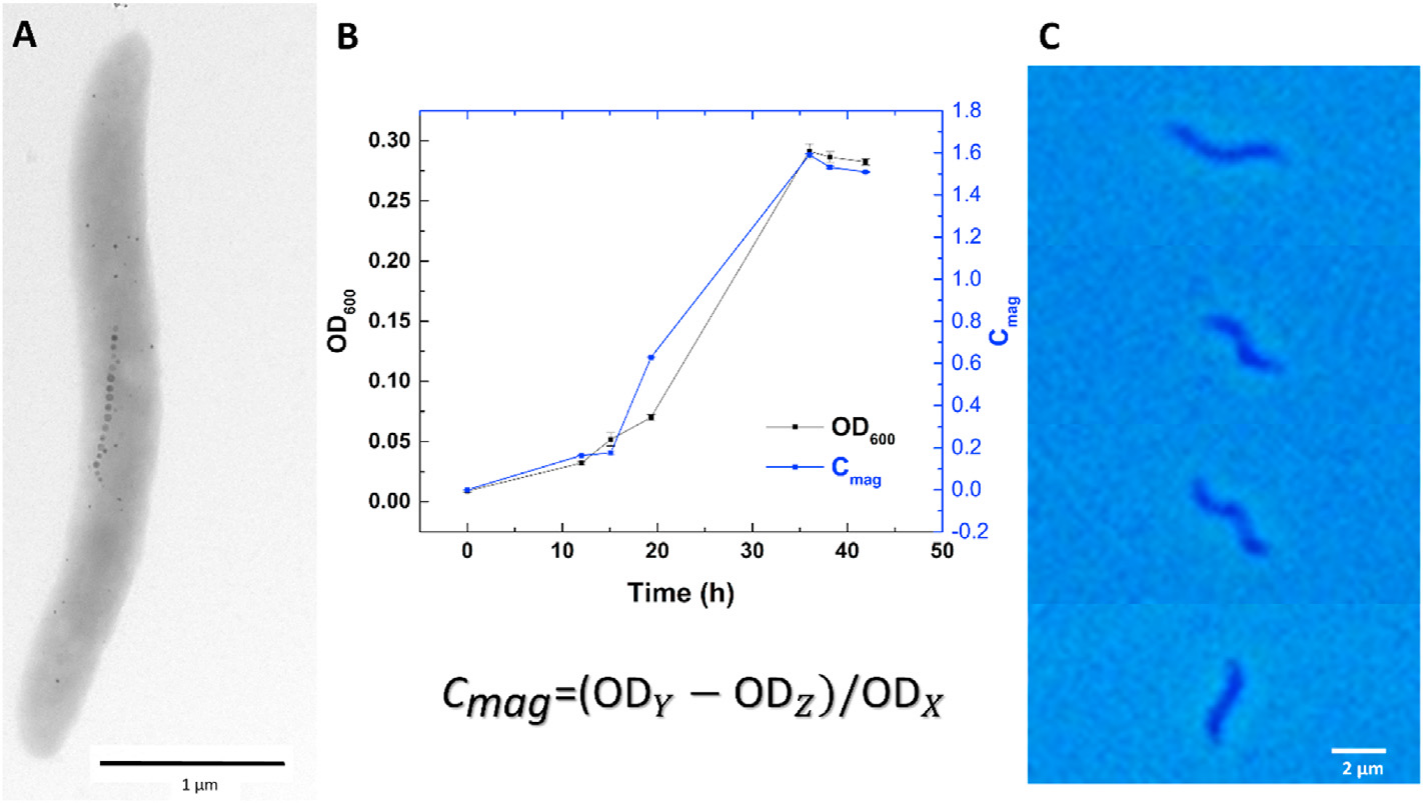
Basic physiological characterization of MSR-1 ​cells: A) TEM image of MSR-1 presenting the classical chain of magnetosomes. B) Combined plot presenting the average growth curve and cell magnetization (*C*_*mag*_) obtained by UV–vis measurements for triplicate measurements of a cultured batch of MSR-1. The optical density (OD) was measured at 600 ​nm (ODx). OD,y,z are the optical densities in the presence of a y and z magnetic fields. C) Optical image displaying the MTB shapes observed to be used as reference.

We used the strain MSR-1 as a model MTB, as this species is one of the most studied MTB. Fig. 1 shows a fully grown MTB imaged with TEM with the typical black points indicating magnetosome particles (Fig. 1A) as well as a growth curve (Fig. 1B) and the semi-quantitative characterization of the magnetic properties by *C*_*mag*_ [31]. These *C*_*mag*_ values are obtained from the UV-VIS measurements and are directly proportional to cells' magnetization. C_mag_ is a relative value and depends on bacterial concentration and cellular length. To obtain a C_mag_ value from UV-VIS measurements, a spectrophotometer equipped with two sets of Helmotz coils, which are perpendicular one to another, is used. The Optical Density (OD) at 600 ​nm of a given bacterial suspension is first measured without applying any magnetic field. This measure is called ODx. Next, bacterial suspension's OD is measured when a magnetic field is applied in a y direction (ODy). Finally, bacterial suspension's OD is measured when a magnetic field is applied in a z direction (ODz) which is perpendicular to the y direction. C_mag_ is calculated using the following formula $\frac{ODy-ODz}{ODx}$ . Therefore, C_mag_ is an estimation of cellular magnetization (given that the difference ODy-ODz will depend on this parameter) normalized to cell density (ODx). From the growth curve, the generation or doubling time is calculated to be around 8 ​h for different batches, in accordance with reported literature data [32,33]. It can be observed that after the complete formation of the magnetosomes, the *C*_*mag*_ stays constant. It is essential to highlight that when one needs to compare different batches with this method, special care has to be taken to cultures inoculation: the cultures have to be inoculated at low cellular density (e.g., [OD in the range of 0.009–0.02] in this case) to ensure enough bacterial divisions and therefore incorporation of the modified amino acid 3-azido-d-alanine hydrochloride in bacterial cell wall. The typical MTB shapes observed from optical microscopy are presented in Fig. 1 and are to be taken as a reference for the following analysis.

**Fig. 2:**
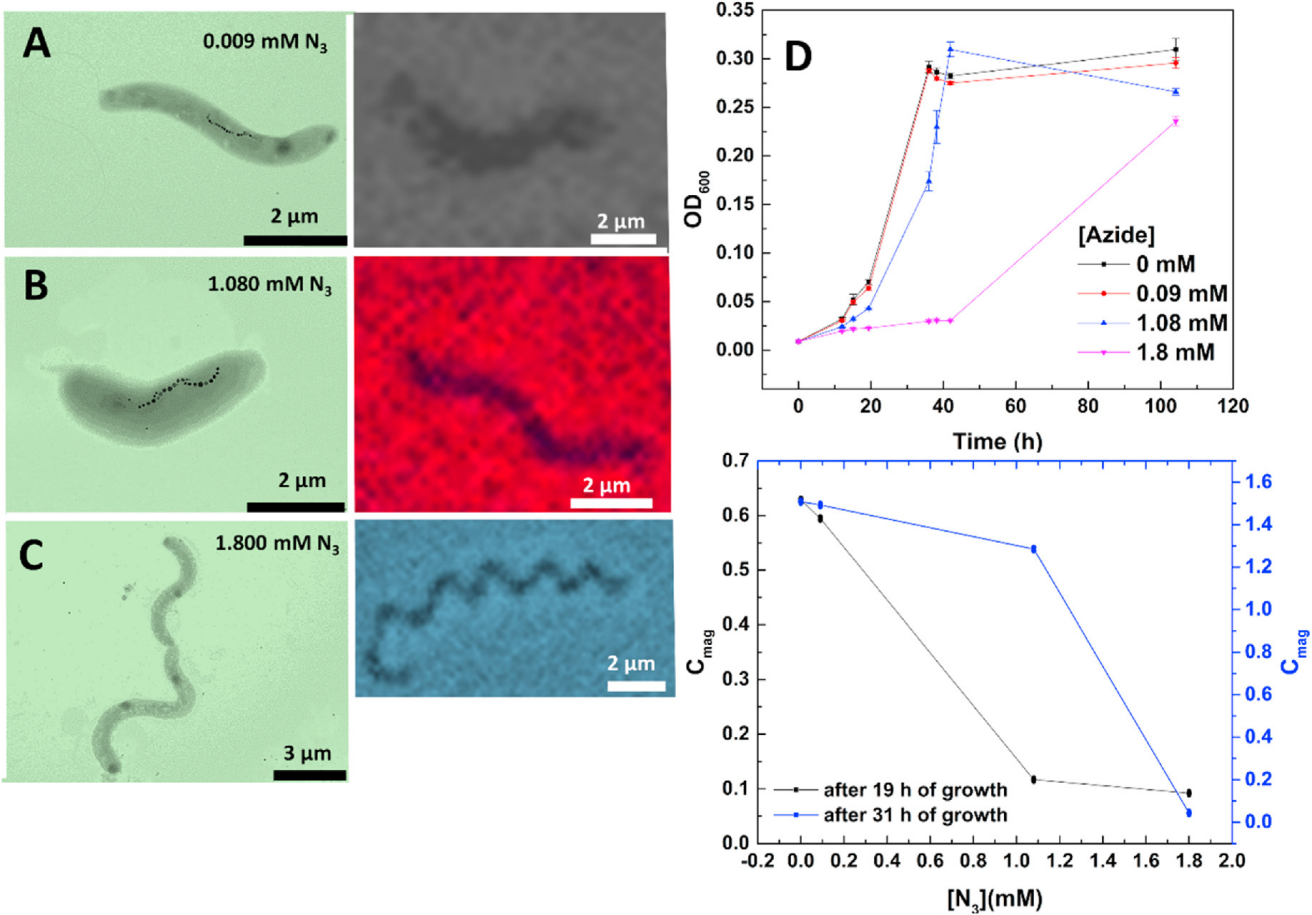
Characterization of the incorporation of the modified amino acid 3-azido-d-alanine hydrochloride in MSR-1. A) - E) show the TEM and optical images of MSR-1 grown in the presence of 0.009 ​mM, 1.080 ​mM and 1.800 ​mM modified amino acid. The plots in D, show the growth curve (top) for MSR-1 in the presence of the different concentrations of the modified amino acid and C_mag_ changes (bottom) for all concentrations after 19 and 31 ​h of growth. All plots are the average of triplicate measurements and are plotted with the standard error as error bar.

We next moved to the first step of our process, corresponding to the incorporation of the azide groups. MSR-1 ​cells were grown in 3 different concentrations of the modified amino acid 3-Azido-d-alanine: 0.009 ​mM, 1.080 ​mM, and 1.800 ​mM. The TEM measurements together with the corresponding optical image for all three cases are shown in Fig. 2A-C. The TEM hints that the number of cells with magnetosome chains decreases upon increased modified amino acid and even more, the long cells do not show any magnetosome production. The growth curve shown at the top part of Fig. 2D, indicates that for both 1.08 and 1.800 ​mM there is a clear delay in growth. The respective doubling times were calculated to be 6.69 ​± ​0.15 ​h for 0.009 ​mM of azide, 7.048 ​± ​0.23 ​h for 1.080 ​mM of azide and 9.08 ​± ​0.06 ​h for 1.800 ​mM of azide. A non-parametrical statistical test was done (Kruskal-Wallis test) obtaining a p-value of 0.36 with alpha ​= ​0.05 and therefore the obtained doubling times cannot be considered significantly different. Analysis of *C*_*mag*_ evolution during growth showed that it decreases upon increasing modified amino acid concentration (Fig. 2D). This decrease seems to be stronger during the exponential phase. To verify if this decrease in *C*_*mag*_ could be related to the cells being stressed upon the rise of modified amino acid concentration, the shape of the cells was analyzed by optical microscopy. Typical forms found for all 3 cases are presented beside the TEM images respectively, and cell shape changes are observed. For 1.080 ​mM many cells stop dividing and are much larger, indicating physiological stress. It is worth noticing that for all three cases, the cells were still motile, as observed from the videos in S1, S2, and S3.

**Fig. 3:**
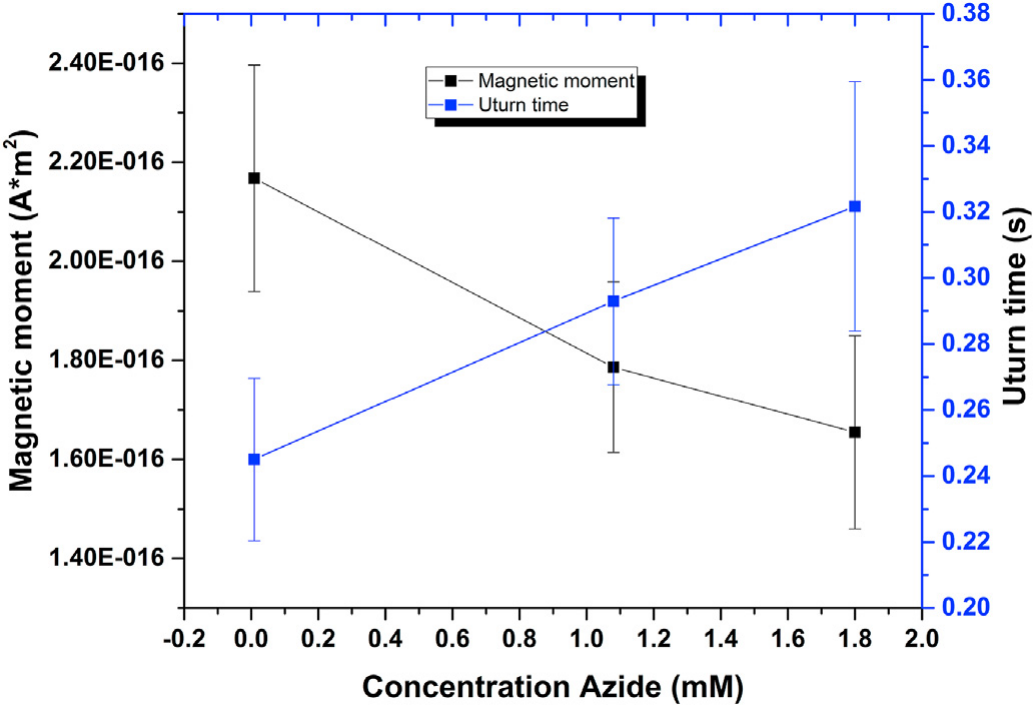
U-turn determination of *Camag* for MSR-1. U-turn time and cell magnetization for MSR-1 grown in different concentrations of modified amino acid.

We further performed U-turn analyses to assess cell magnetization quantitatively. For this analysis we exclude all long cells as no magnetosome were observed from the TEM measurements thus no response with external magnetic fields is expected. We therefore considered the average size found from 40 TEM images, namely length L ​= ​3.46 ​± ​0.24 ​μm and width of W ​= ​0.54 ​± ​0.11 ​μm for the U-turn analysis. Briefly, the trajectory of a motile cell is recorded in the case in which an applied magnetic field switches direction. This switching makes the cells do a U-turn and change direction. It is possible to calculate cell magnetization from the time it takes the cell to make the U-turn ([34] and method section). In Fig. 3, cell magnetization and U-turn time for all 3 concentrations are shown, and the apparent decrease of cell magnetization upon modified amino acid concentration shown in Fig. 2D is again observed. The same Figure is shown in Fig. S1A together with the detailed graphs S1B–S1D necessary to calculate the U_turn_. Fig. S1B, shows a typical U-turn trajectory a single MSR-1 ​cell performs upon external magnetic field switching. Fig. S1C shows the motion along a single direction (Y), displaying the change in orientation upon the applied magnetic field; the peak indicates this change in direction. Fig. S1D shows the derivative of S1C and transforms the peak into a small step used to extract the U-turn time more precisely.

**Fig. 4:**
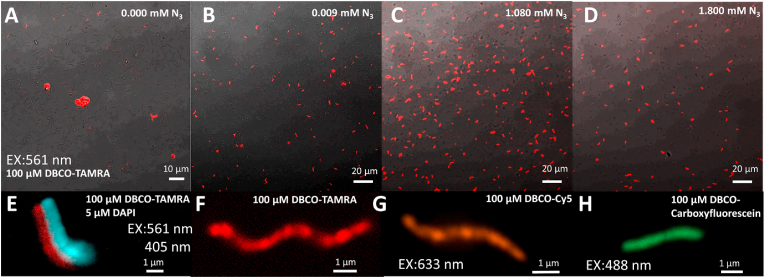
Fluorescent chemical compounds attachment by copper-free click-chemistry. A-D) 100 ​μM of DBCO-TAMRA attachment for MSR-1 grown in 4 different modified amino acid concentrations 0, 0.009, 1.080 and 1.800 ​mM, respectively. E) DNA staining by DAPI in conjunction with DBCO-TAMRA with double excitation source 405 ​nm for DAPI and 561 ​nm for TAMRA. F–H) A single-cell image for MSR-1 with DBCO-PEG4-FLUOR 545; DBCO-Cy5 and DBCO-dPEG ®12-carboxyfluorescein.

The presence of the biorthogonal azide group was verified, and for this, the batches grown with no modified amino acids, i.e., zero (control) and the same three concentrations, i.e. 0.009 ​mM, 1.080 ​mM, and 1.800 ​mM of the modified amino acid were mixed with 100 ​μM of the fluorescent biomolecule DBCO-TAMRA. The samples were analyzed by confocal imaging, and the images are shown in Fig. 4A-D. The control shows some clusters of fluorescent biomolecules, but almost no MTB tagging (Fig. 4A). The amount of MSR-1 tagged increases from 0.009 ​mM to 1.080 ​mM but decreases for 1.80 ​mM. A semi-quantification was done with a costum python script (see method section). The script extracts the total amount of fluorescence labeled MTB and the total amount of MTB in the images. A relative error is then defined as total amount of MTB minus number of tagged MTB divided by the total amount of MTB and converted to percentage by multiplying by 100. This relative error is considered as a parameter to compare the tagging efficiency. Please note that this represents a minimum of tagging efficiency as it includes all MTB present in the images including those slightly out of focus that could potentially be tagged but not observed. We used 5 images for each azide concentration with over 100 MTB in each image and averaged the relative error. In Table 1, the tagging efficiency for each used azide concentration is indicated.

**Table 1:** Fluorescent tagging efficiency obtained for each azide concentration used.

Azide concentration (mM)	Tagging efficiency (%)
0.009	(5.30 ​± ​0.01)
1.080	(24.11 ​± ​0.02)
1.800	(17.57 ​± ​0.01)

The increased labeled number of MSR-1 ​cells observed for 1.080 ​mM suggests that this concentration is the best tested concentration (among those probed) allowing to have bacterial azide incorporation while retaining cell magnetization. Therefore, we considered this concentration and used it to exemplify the versatility of our approach with other tagging methods. Optical images with different functionalization are shown below: A single cell labeled with DAPI and TAMRA (Fig. 4E and movie S3), TAMRA (Fig. 4F), Cy5 (Fig. 4G) and carboxyfluorescein (Fig. 4H).

Supplementary data related to this article can be found at https://doi.org/10.1016/j.mtbio.2023.100587.

The following is the supplementary data related to this article:

Movie S3 shows motile MSR-1 grown with 0.1 mM of modified amino azide with a +/-3.5mT magnetic field applied along the vertical, y, direction, switching every 2 sec once the field is activated Movie S3

**Fig. 5:**
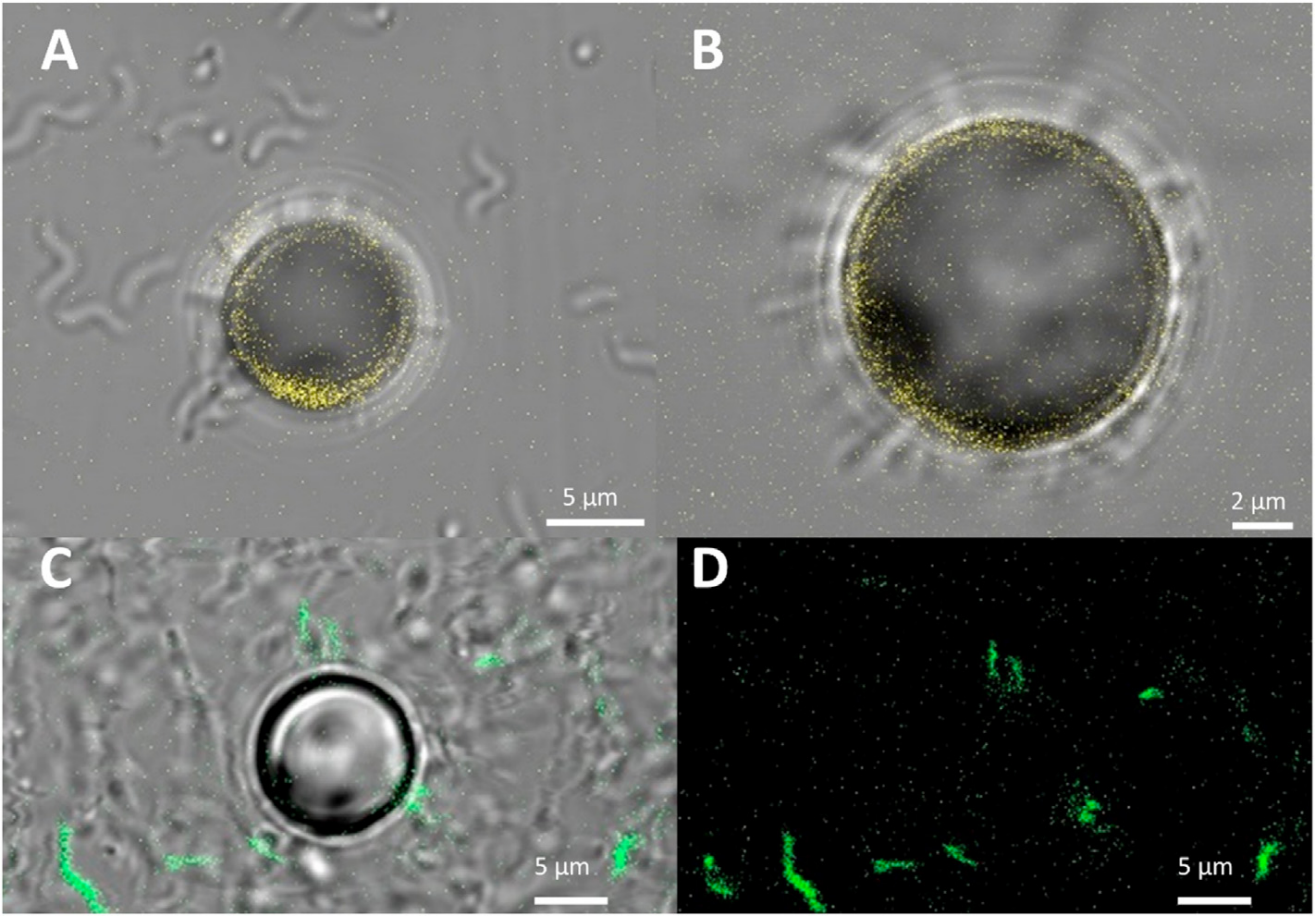
A) and B) show the optical image of the 10 μm Micromer® beads with N_3_ modified MSR-1 attached to the surface. The Micromer® beads show fluorescence at n excitation wavelength of 405 ​nm. Similarly C) shows combined fluorescence (excitation wavelength of 488 ​nm) and bright field MSR1-GGFP-N_3_ attached to the beads and D) only the GFP Fluorescence from MSR-1.

We analyzed the attachment to relatively large, 10 ​μm, Micromer®-DBCO beads. In Fig. 5 A) an optical image is presented showing the effective attachment of N_3_ modified MSR-1 to the Micromer®-DBCO beads in different configurations and in B) the N_3_ modified MSR-1 are shown to attach homogenously over the surface. Fig. 5 C) shows a combined optical and fluorescence image of the same Micromer®-DBCO beads in the presence of N_3_ modified GFP tagged MSR-1, The blurred background are swimming MSR-1. Fig. 5 D) shows the GFP fluorescence solely demonstrating the attachment on the beads surface. We checked if the beads with attached bacteria presented any motion or magnetic response and verified that there was none. Movie S5 indicates that the beads with the attached MSR-1 do not present any directional motion while movie S6 shows that even with high number of attached MSR-1 there still is no motion. In Movie 7 we used a custom magnetic microscope [26] to apply a 3 ​mT rotating magnetic field and show the lack of response form the bead together with the rotating MSR-1.

Supplementary data related to this article can be found at https://doi.org/10.1016/j.mtbio.2023.100587.

The following are the supplementary data related to this article:

Movie S5 shows 10μm Micromer® beads with N_3_ modified MSR-1 attached to the surface with different configuration. Movie S5

Movie S6 shows 10μm Micromer® beads with N_3_ modified MSR-1 homogenously attached to the surface. Movie S6

Movie S7 shows 10 μm Micromer® beads with N_3_ modified MSR-1 attached to the surface with an 3 ​mT rotating magnetic field. Movie S7

Compared with previous works, while assuming that the available surface of the bacteria is the same between each culture batch of a given strain, the immobilization success rate depends on two things: 1) the yield of bonding and 2) the yield of healthy bacteria. The click chemistry is bio-orthogonal and compatible with different biomolecules. Nevertheless, as shown here, the azide group incorporation through metabolic labeling can influence the physiological properties of MTB, specifically magnetosome production. It remains to be seen if a similar effect occurs when bacteria are genetically engineered. For example, overproduction of magnetosomes has been observed previously by genomic amplification [35].

For nanoparticles, several studies have shown a higher immobilization yield through click chemistry compared to carbodiimide (EDC/NHS chemistry) [36,37]. Carbodiimide was previously used to attach nanoliposomes on MTB (specifically to the strain *Manetococcus marinus* (MC-1) [9]). The chemical conjugation of liposomes functionalized with reactive groups (–COOH) allows the covalent binding to primary amino groups (–NH_2_) intrinsic to bacterial cell membrane proteins. Another approach that bonds cargo to the primary groups of a MTB, more specifically to *Magnetospirilium magneticum* (AMB-1), was presented [11]. The authors used an MTB/PEG–biotin complex, corresponding to a covalent bond formation between bacteria and biotin–PEG–NHS polymer. In that case, the main mechanism also includes a nucleophilic attack as in the case of EDC/NHS where NHS esters are formed and attached to the primary amino groups leaving the biotin-PEG-NHS on the bacteria surface and allowing further functionalization by streptavidin-biotin interaction. A third approach introduced immobilized Indocyanine green nanoparticles (INPs) functionalized with maleimide groups on AMB-1 [10]. The INPs were chemically conjugated to activated sulfhydryl on the surface of AMB-1 bacteria by Michael addition reaction. Overall, just looking at an individual cell, the amount of cargo that can be immobilized will then depend on the available functional groups, amino, activated sulfhydryl (activated disulfide surface proteins) or in our case the amount of azide groups and of course the affinity and selectivity of the bonding at hand. Click chemistry has the advantage of selectivity and therefore of yield when compared to the other immobilization techniques. Another advantage of the present work is that we do not need to activate surface groups. Still in all cases, many steps are needed to add or have available chemical groups and attach the cargo. The click chemistry has the advantage, compared to other covalent immobilization techniques used, of the bio-orthogonality and selectivity of the reactions. However, it still presents the disadvantage that to achieve the immobilization of specific groups on the biomolecule and bacterial surface, several reaction steps must be carried out on the respective units to prepare them for the click reaction. The problem of having several reaction steps could be partially overcome by, for example, bacterial genetic engineering. Nevertheless, as demonstrated in this work, care must be taken to find the right conditions to maintain the MTB's general physiological properties. In this work we also show the effective attachment to 10 ​μm Micromer®-DBCO beads. However, this attachment prevents bacterial motion and magnetic response. We attribute this lack of response to the size of the beads as they precipitate and get fixed to the glass slide adding additional resistance to external forces. We envision that smaller particles should allow directional motion stirred by the MTB as shown in previous work [9].

The copper-free strain-promoted azide-alkyne cycloaddition is one of the most widely employed in bio-orthogonal chemistry and, as indicated previously, has been employed by other bacteria to attach cargo [25]. However, in the mentioned work, the physiological properties of the bacterium *E. coli strain Seattle 1946* used were not studied. The binding efficiency was assessed on fixed bacteria, and the motility after modification was assumed to have negligible changes compared to the non-modified bacteria referring to a previous work that indicated a 65% decrease in speed at the highest bacteria motility of a bacterium with cargo compared to a free bacterium [38]. It was not experimentally tested, nor was the shape of the bacterium analyzed upon different azide concentrations to see if any physiological stress builds up. Although in our work, we also do not observe a clear change in speed, the MTB growth is clearly affected showing difference in shape and even effects upon the biosynthesis of magnetosomes. Fixating bacteria and inhibiting their growth are most likely the reasons, as the physiological stress build-up was not observed previously.

## 4 Conclusion

Copper-free click chemistry was successfully proven by mixing DBCO- TAMRA with MTB where a modified amino acid was integrated into the periplasm. The best conditions for the azide incorporation were then used to show its compatibility with other tagging methods such as DAPI and biomolecules by single cell confocal imaging. In addition, we exemplarily have attached 10 ​μm DBCO-Polystyrene beads. However, due to their dimension, the beads sediment to the glass and get fixed. Despite the fixation, many MTB are capable of attaching to the beads using the protocol we have developed. We observe that to maintain bacterial mobility once attached, smaller particles should be used. These findings open the possibilities of designing MTB functionalized through click chemistry with any biomolecules and are extremely important for future MTB bots designs or MTB applied to bio-sensing, energy or environmental applications where functionalized MTB are envisioned for increased performance [39].

## Credit author statement

**Paul Eduardo David Soto Rodriguez**: Conceptualization, Methodology Software and validation, Writing-original. **Mila Sirinelli-Kojadinovic**: Data curation, Methodology, Formal analysis, Validation and writing –review & editing. **Maximilien Rouzaud**: Data curation and visualization; **Damien Faivre**: Supervision, writing –review & editing, Resources and funding acquisition

## Declaration of competing interest

The authors declare that they have no known competing financial interests or personal relationships that could have appeared to influence the work reported in this paper.

## Data Availability

Data will be made available on request.

## References

[1] Soto F., Chrostowski R. (2018). Frontiers of medical micro/nanorobotics: in vivo applications and commercialization perspectives toward clinical uses. Front. Bioeng. Biotechnol..

[2] Zhou H., Mayorga-Martinez C.C., Pané S., Zhang L., Pumera M. (2021). Magnetically driven micro and nanorobots. Chem. Rev..

[3] Coene A., Leliaert J. (2022). Magnetic nanoparticles in theranostic applications. J. Appl. Phys..

[4] Martel S. (2016). Swimming microorganisms acting as nanorobots versus artificial nanorobotic agents: a perspective view from an historical retrospective on the future of medical nanorobotics in the largest known three-dimensional biomicrofluidic networks. Biomicrofluidics.

[5] Sahari A., Traore M.A., Stevens A.M., Scharf B.E., Behkam B. (2014). Toward development of an autonomous network of bacteria-based delivery systems (BacteriaBots): spatiotemporally high-throughput characterization of bacterial quorum-sensing response. Anal. Chem..

[6] Mostaghaci B., Yasa O., Zhuang J., Sitti M. (2017). Bioadhesive bacterial microswimmers for targeted drug delivery in the urinary and gastrointestinal tracts. Adv. Sci..

[7] Schauer O., Mostaghaci B., Colin R., Hürtgen D., Kraus D., Sitti M., Sourjik V. (2018). Motility and chemotaxis of bacteria-driven microswimmers fabricated using antigen 43-mediated biotin display. Sci. Rep..

[8] Bastos-Arrieta J., Revilla-Guarinos A., Uspal W.E., Simmchen J. (2018). Bacterial biohybrid microswimmers. Front. Robot. AI..

[9] Felfoul O., Mohammadi M., Taherkhani S., de Lanauze D., Zhong Xu Y., Loghin D., Essa S., Jancik S., Houle D., Lafleur M., Gaboury L., Tabrizian M., Kaou N., Atkin M., Vuong T., Batist G., Beauchemin N., Radzioch D., Martel S. (2016). Magneto-aerotactic bacteria deliver drug-containing nanoliposomes to tumour hypoxic regions. Nat. Nanotechnol..

[10] Xing J., Yin T., Li S., Xu T., Ma A., Chen Z., Luo Y., Lai Z., Lv Y., Pan H., Liang R., Wu X., Zheng M., Cai L. (2020). Sequential magneto-actuated and optics-triggered biomicrorobots for targeted cancer therapy. Adv. Funct. Mater..

[11] Chaturvedi R., Kang Y., Eom Y., Torati S.R., Kim C. (2021). Functionalization of biotinylated polyethylene glycol on live magnetotactic bacteria carriers for improved stealth properties. Biology.

[12] Lefevre C.T., Bazylinski D.A. (2013). Ecology, diversity, and evolution of magnetotactic bacteria. Microbiol. Mol. Biol. Rev..

[13] Lefèvre C.T., Bennet M., Landau L., Vach P., Pignol D., Bazylinski D.A., Frankel R.B., Klumpp S., Faivre D. (2014). Diversity of magneto-aerotactic behaviors and oxygen sensing mechanisms in cultured magnetotactic bacteria. Biophys. J..

[14] Kuzajewska D., Wszołek A., Żwierełło W., Kirczuk L., Maruszewska A. (2020). Magnetotactic bacteria and magnetosomes as smart drug delivery systems: a new weapon on the battlefield with cancer?. Biology.

[15] Mériaux S., Boucher M., Marty B., Lalatonne Y., Prévéral S., Motte L., Lefèvre C.T., Geffroy F., Lethimonnier F., Péan M., Garcia D., Adryanczyk-Perrier G., Pignol D., Ginet N. (2015). Magnetosomes, biogenic magnetic nanomaterials for brain molecular imaging with 17.2 T MRI scanner. Adv. Healthcare Mater..

[16] Kraupner A., Eberbeck D., Heinke D., Uebe R., Schüler D., Briel A. (2017). Bacterial magnetosomes – nature's powerful contribution to MPI tracer research. Nanoscale.

[17] Makela A.V., Schott M.A., Madsen C.S., Greeson E.M., Contag C.H. (2022). Magnetic particle imaging of magnetotactic bacteria as living contrast agents is improved by altering magnetosome arrangement. Nano Lett..

[18] Devaraj N.K., Finn M.G. (2021). Introduction: click chemistry. Chem. Rev..

[19] Worrell B.T., Malik J.A., Fokin V.V. (2013). Direct evidence of a dinuclear copper intermediate in Cu(I)-Catalyzed azide-alkyne cycloadditions. Science.

[20] Li L., Zhang Z. (2016). Development and applications of the copper-catalyzed azide-alkyne cycloaddition (CuAAC) as a bioorthogonal reaction. Molecules.

[21] Chin J.W., Santoro S.W., Martin A.B., King D.S., Wang L., Schultz P.G. (2002). Addition of *p* -Azido- l -phenylalanine to the Genetic Code of *Escherichia c oli*. J. Am. Chem. Soc..

[22] Reddington S., Watson P., Rizkallah P., Tippmann E., Jones D.D. (2013). Genetically encoding phenyl azide chemistry: new uses and ideas for classical biochemistry. Biochem. Soc. Trans..

[23] Chang P.V., Prescher J.A., Sletten E.M., Baskin J.M., Miller I.A., Agard N.J., Lo A., Bertozzi C.R. (2010). Copper-free click chemistry in living animals. Proc. Natl. Acad. Sci. U.S.A..

[24] Moreno V.M., Álvarez E., Izquierdo–Barba I., Baeza A., Serrano–López J., Vallet–Regí M. (2020). Bacteria as nanoparticles carrier for enhancing penetration in a tumoral matrix model. Adv. Mater. Interfac..

[25] Heyen U., Schüler D. (2003). Growth and magnetosome formation by microaerophilic Magnetospirillum strains in an oxygen-controlled fermentor. Appl. Microbiol. Biotechnol..

[26] Bennet M., McCarthy A., Fix D., Edwards M.R., Repp F., Vach P., Dunlop J.W.C., Sitti M., Buller G.S., Klumpp S., Faivre D. (2014). Influence of magnetic fields on magneto-aerotaxis. PLoS One.

[27] Dardagan N., Brdanin A., Dzigal D., Akagic A. (2021). 2021 IEEE 30th International Symposium on Industrial Electronics (ISIE).

[28] Pierce C.J., Mumper E., Brown E.E., Brangham J.T., Lower B.H., Lower S.K., Yang F.Y., Sooryakumar R. (2017). Tuning bacterial hydrodynamics with magnetic fields. Phys. Rev. E..

[29] Leão P., Le Nagard L., Yuan H., Cypriano J., Da Silva–Neto I., Bazylinski D.A., Acosta–Avalos D., Barros H.L., Hitchcock A.P., Lins U., Abreu F. (2020). Magnetosome magnetite biomineralization in a flagellated protist: evidence for an early evolutionary origin for magnetoreception in eukaryotes. Environ. Microbiol..

[30] OriginPro (2016).

[31] Schüler D., Uhl R., Bäuerlein E. (1995). A simple light scattering method to assay magnetism in *Magnetospirillum gryphiswaldense*. FEMS (Fed. Eur. Microbiol. Soc.) Microbiol. Lett..

[32] Riese C.N., Uebe R., Rosenfeldt S., Schenk A.S., Jérôme V., Freitag R., Schüler D. (2020). An automated oxystat fermentation regime for microoxic cultivation of Magnetospirillum gryphiswaldense. Microb. Cell Factories.

[33] Kolinko I., Jogler C., Katzmann E., Schüler D. (2011). Frequent mutations within the genomic magnetosome island of Magnetospirillum gryphiswaldense are mediated by RecA. J. Bacteriol..

[34] Esquivel D.M.S., Barros H.G. de P.L. de (1985). http://inis.iaea.org/search/search.aspx?orig_q=RN:17012441.

[35] Lohße A., Kolinko I., Raschdorf O., Uebe R., Borg S., Brachmann A., Plitzko J.M., Müller R., Zhang Y., Schüler D. (2016). Overproduction of magnetosomes by genomic amplification of biosynthesis-related gene clusters in a magnetotactic bacterium. Appl. Environ. Microbiol..

[36] Thorek D.L.J., Elias ew R., Tsourkas A. (2009). Comparative analysis of nanoparticle-antibody conjugations: carbodiimide versus click chemistry. Mol. Imag..

[37] Bolley J., Guenin E., Lievre N., Lecouvey M., Soussan M., Lalatonne Y., Motte L. (2013). Carbodiimide versus click chemistry for nanoparticle surface functionalization: a comparative study for the elaboration of multimodal superparamagnetic nanoparticles targeting α _v_ β _3_ integrins. Langmuir.

[38] Traore M.A., Damico C.M., Behkam B. (2014). Biomanufacturing and self-propulsion dynamics of nanoscale bacteria-enabled autonomous delivery systems. Appl. Phys. Lett..

[39] Ebrahimi N., Bi C., Cappelleri D.J., Ciuti G., Conn A.T., Faivre D., Habibi N., Hošovský A., Iacovacci V., Khalil I.S.M., Magdanz V., Misra S., Pawashe C., Rashidifar R., Soto–Rodriguez P.E.D., Fekete Z., Jafari A. (2021). Magnetic actuation methods in bio/soft robotics. Adv. Funct. Mater..

