# Supplementary material for: Azide click chemistry on magnetotactic bacteria: A versatile technique to attach a cargo
Source: Mater Today Bio. 2023 Feb 23;19:100587. doi: 10.1016/j.mtbio.2023.100587 (PMC9999208; doi:10.1016/j.mtbio.2023.100587)
Supplement: Multimedia component 1 [file mmc1.docx]

Supporting information

**Azide click chemistry on magnetotactic bacteria: a versatile technique to attach a cargo**

Authors:

Paul Eduardo David Soto Rodriguez^1^, Mila Sirinelli-Kojadinovic^1^, Maximilien Rouzaud^1^ and Damien Faivre^1*^

^1^ Aix Marseille University, CEA, CNRS, BIAM, 13108 Saint Paul-Lez-Durance, France

*Corresponding author


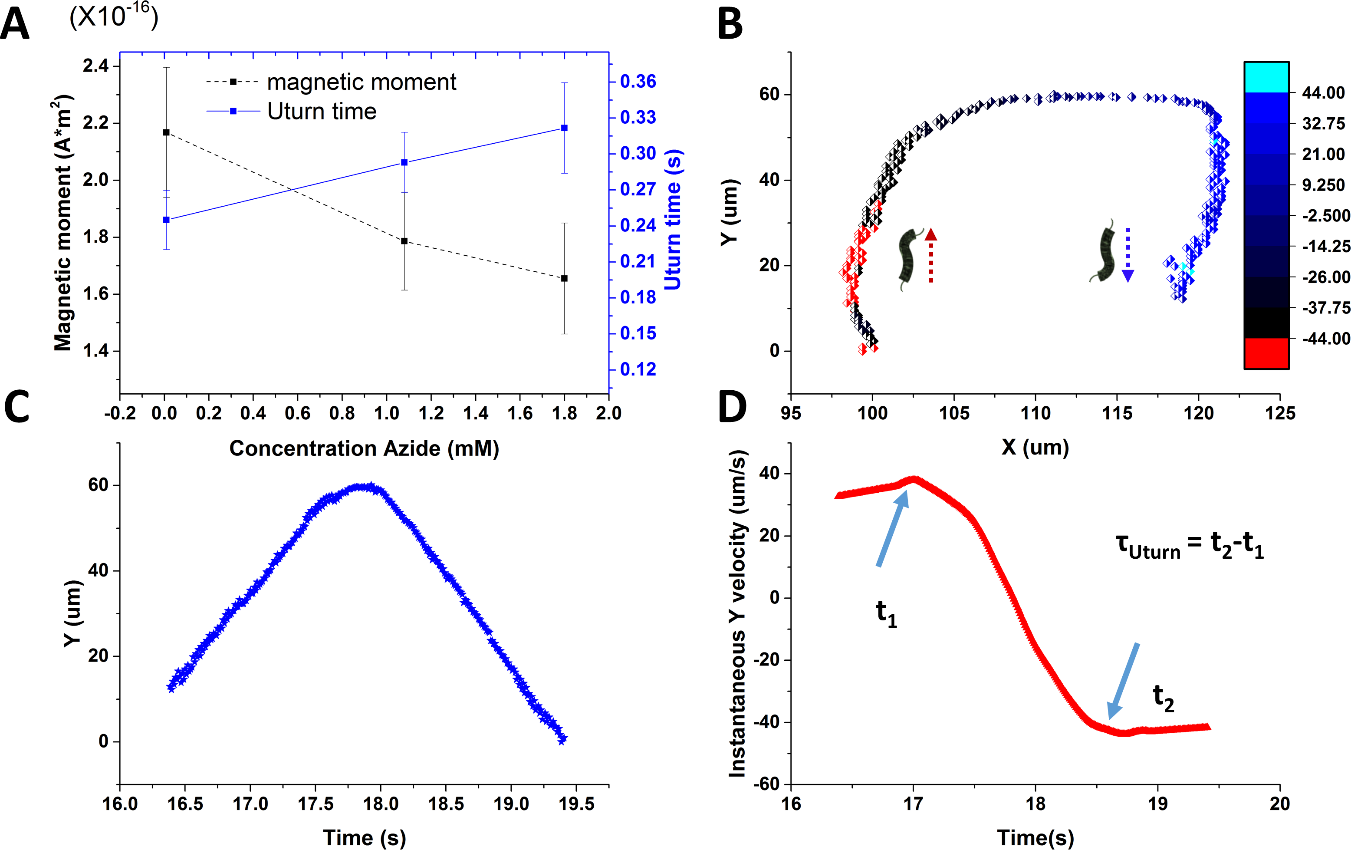


Figure S1: U-turn determination of *Cmag* for MSR-1. A) U-turn time and cell magnetization for MSR-1 grown in different concentrations of modified amino acid. B) Typical U-turn trajectory observed. D) The movement along Y during the U-turn in the function of time. D) The derivative of C) from where the U-turn time ***τ_Uturn_*** is calculated.
